# Ultrahigh Responsivity-Bandwidth Product in a Compact InP Nanopillar Phototransistor Directly Grown on Silicon

**DOI:** 10.1038/srep33368

**Published:** 2016-09-23

**Authors:** Wai Son Ko, Indrasen Bhattacharya, Thai-Truong D. Tran, Kar Wei Ng, Stephen Adair Gerke, Connie Chang-Hasnain

**Affiliations:** 1 Department of Electrical Engineering and Computer Sciences, University of California at Berkeley, Berkeley, California 94720, United States of America

## Abstract

Highly sensitive and fast photodetectors can enable low power, high bandwidth on-chip optical interconnects for silicon integrated electronics. III-V compound semiconductor direct-bandgap materials with high absorption coefficients are particularly promising for photodetection in energy-efficient optical links because of the potential to scale down the absorber size, and the resulting capacitance and dark current, while maintaining high quantum efficiency. We demonstrate a compact bipolar junction phototransistor with a high current gain (53.6), bandwidth (7 GHz) and responsivity (9.5 A/W) using a single crystalline indium phosphide nanopillar directly grown on a silicon substrate. Transistor gain is obtained at sub-picowatt optical power and collector bias close to the CMOS line voltage. The quantum efficiency-bandwidth product of 105 GHz is the highest for photodetectors on silicon. The bipolar junction phototransistor combines the receiver front end circuit and absorber into a monolithic integrated device, eliminating the wire capacitance between the detector and first amplifier stage.

With ever-increasing data rates and integration density in electronic integrated circuits, optical interconnects are becoming a compelling energy-saving choice for high speed, broadband communication links on and between chips. The power consumed at a certain data rate, equivalently expressed as the energy per bit, is heavily dependent on the capacitance of the receiver^1^,^2^. A receiver with low total capacitance *C*_*tot*_ is able to produce a sufficiently high voltage signal *V*_*sig*_ for a reduced number of photons per bit: *V*_*sig*_ = *q* × *n*_*bit*_/*C*_*tot*_. This lowers the minimum link power consumption by reducing both transmitter power - by decreasing *n*_*bit*_: the required number of photons per bit in the link, as well as receiver power necessary to amplify the small voltage signal *V*_*sig*_ to eventually drive CMOS logic. With this goal, there has been significant work on reducing the capacitance of photodetectors with an indirect bandgap Germanium absorber grown on a technologically relevant silicon substrate^3^,^4^,^5^,^6^. Thin 2-dimensional (2D) materials such as graphene and transition metal dichalcogenides are also being explored for fast photodetection due to the possibility of small transit times^7^,^8^. An unresolved hurdle here is that light absorption is extremely weak due to the thin topology and integration with very long waveguides is required to provide any reasonable responsivity, leading to a large device area and high capacitance^9^. Direct bandgap III-V based compound semiconductors, particularly the InP/InGaAs system, have at least an order of magnitude higher absorption coefficient than silicon and germanium, and also permit heterostructures and bandgap engineering for highly functional devices^10^,^11^. Such III-V devices can approach almost complete absorption with a near-wavelength scale volume and enable ultra-low capacitance photodetectors while maintaining excellent quantum efficiency and low dark current. From a link sensitivity analysis^12^, a 100 nm diameter III-V compound semiconductor device would have a self-capacitance as low as 50 aF, potentially allowing low energy on-chip optical links to approach fundamental shot noise limited communication at a bit error ratio of 10^−9^ with merely 20 photons/bit in the link^13^.

Despite the excellent potential to scale down the capacitance of III-V compound semiconductor detectors, an optical link with off-chip III-V devices suffers from the parasitic capacitance of the electrical wire connecting the detector to the next stage. Attempts to overcome this have been made by bringing the photonic chips close to the electronic circuits using, for instance, through-oxide vias^14^,^15^. Nonetheless, even a 10 *μm* separation adds 2 fF of capacitance^16^, which translates into a requirement for additional link power to maintain the same signal to noise ratio. This is particularly severe if the capacitance of the photodetector is much lower than the parasitic wire capacitance.

To address this issue, it is important to integrate gain directly into the photodetector and improve sensitivity. This could also be viewed as an intimate integration of the first amplifier stage with the photodetector, thus eliminating the wire capacitance altogether. Several approaches have been attempted, including Si/Ge and In(Al,Ga)As avalanche photodiodes^17^,^18^,^19^,^20^, field effect phototransistors^21^,^22^ and bipolar junction phototransistors^10^,^23^,^24^,^25^,^26^,^27^,^28^,^29^,^30^,^31^. Avalanche photodiodes with high gain-bandwidth product have been demonstrated^17^,^18^,^19^. However, the high gain does not necessarily translate into better sensitivity due to the fundamental excess noise associated with the avalanche process^32^. Additionally, APDs require a thermal compensation mechanism due to the sensitive dependence of impact ionization on temperature^33^,^34^,^35^ and a high reverse bias voltage (10 V or higher), both of which are undesirable for densely integrated chip-scale applications. Approaches based on nanowire photoconductive devices also show very large gain due to long carrier lifetimes^36^,^37^,^38^. On the other hand, the long lifetime limits the speed of these devices – with response times on the order of milliseconds to 100 seconds, leading to insufficient gain bandwidth product^37^,^39^. Phototransistors (both BJT and FET based) are expected to be highly sensitive due to the absence of multiplication noise and the gain bandwidth product can be increased by simply scaling the device size down, in analogy with transistor cutoff frequency^40^. A phototransistor is in fact expected to be even more sensitive than a p-i-n photodiode and transistor combination due to the absence of the wire capacitance^41^,^42^. The bipolar junction transistor is preferred due to a significantly higher transconductance compared to a field effect device. The transconductance for the BJT is linear and is determined by the thermal voltage of 25.9 mV rather than the ~100 mV field effect transistor overdrive voltage. This allows a higher gain-bandwidth product to be reached at the same DC collector current^43^. While promising gain-bandwidth products have been achieved using III-V based phototransistors with highly scaled geometries, the small size leads to deficient photon absorption^23^,^26^,^27^,^28^,^30^. Additionally, the high lattice mismatch of InP and GaAs with silicon leads to defective material unsuitable for devices. In this work, a bipolar transistor gain mechanism is implemented in a direct bandgap III-V semiconductor material to achieve high intrinsic photoresponse, as well as high gain-bandwidth product in an integrated optoelectronic receiver grown and fabricated directly on silicon with CMOS compatibility.

## Results

### Regrown Nanopillar Device Growth and Fabrication on Silicon

Despite the high absorption and low dark current of III-V detectors, applications have been challenged by the difficulty of integrating with large scale CMOS technology and silicon based read out circuitry. In this work, a major technological hurdle has been overcome by monolithically growing optoelectronic quality III-V material on silicon at a substantially lower growth temperature than the ~800 °C growth temperature that is usual for conventional III-V epitaxy. The InP photoBJT was synthesized on silicon using a novel metastable nanopillar growth mode. Single crystalline InP nanopillars were grown under conditions demonstrated to be CMOS post-process compatible^44^. The bulk of the pillar was defect free in spite of a large crystal lattice mismatch of 8%. The strain due to lattice mismatch is locally relaxed through arrays of misfit dislocations at the base of the InP nanopillar, right at the Silicon interface^45^. TEM images confirm pristine Wurtzite phase in the body of the pillar.

The growth is in a radial manner, with tunable material composition and doping possible in the core-shell direction, using which p-i-n junction diode devices have already been demonstrated^46^,^47^. As opposed to an axial distribution of dopants, the core-shell junction geometry is highly beneficial in terms of minimizing surface exposure of the minority carriers and leads to a far reduced dark current for the base-emitter junction^48^. Another benefit of the core-shell or wrap-around geometry, in analogy with waveguide integrated photodetectors, is that it decouples the light propagation direction from the carrier transport direction, thus allowing the photogenerated carriers to diffuse to the p-n-p junction within a short distance^4^,^49^. This facilitates a rapid photoresponse and high responsivity-bandwidth product.

*In-situ* dopant incorporation with nanometer scale precision eliminates the requirement for implantation and diffusion based doping. The resulting sharp electrical junctions are important for obtaining rapid device operation. The base transit time is kept low by having a thin base with electrical width of ten nanometers or less. In addition, the collector-base junction depletion width is kept thin by using a high base and collector doping, reducing the collector transit time. The base doping was measured in an isotype n-doped pillar with the same dopant precursor flow rate using the Burstein-Moss shift in photoluminescence peak and estimated to be approximately 10^18^ *cm*^−3^ (Ref.  48).

**Figure 1:**
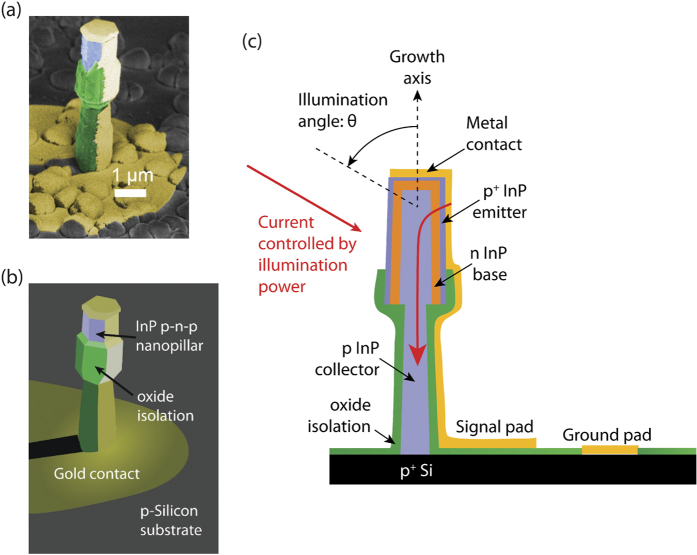
Device geometry. Scanning electron microscope image (**a**) and 3d schematic (**b**) showing the device structure. The sub-micron diameter device is directly grown on Silicon and fabricated by isolating the top contact from the substrate using an oxide sleeve. Further fabrication details are in supplementary section I and II. Half of the device is exposed for angled light illumination, and the radio frequency signal is collected using a G-S high speed probe. The device cross section (**c**) shows that the active region is in the regrown part of the pillar. When light is shone on the device, carriers are injected into the thin base region (orange), and the device can be rapidly turned on and off. The illumination angle was chosen to be θ = 60 degrees from the growth axis for the experiments, but the device absorption is relatively angle insensitive.

On directly growing a p-n-p junction device on silicon, the outer p-shell, which is heavily doped as the emitter, comes into contact with the p-type silicon substrate, thus shunting the actual device junction (see Supplementary Fig. S1-f). Therefore, it is critical to electrically isolate the outer layers from the substrate. This challenge was also encountered for p-i-n junction fabrication and alleviated using a carefully engineered regrown junction. The process is illustrated in Supplementary Fig. S1a–d and summarized here. Once the primary growth is completed, an amorphous Silicon sleeve is defined on the lower section of the nanopillars. After this, the regrowth occurs selectively on the upper section in a core-shell manner and at a slightly elevated growth temperature compared to the original growth. The active region is completely in the regrown portion. In this manner, it can be seen that the current is directed through the p-n-p junction as desired (Fig. 1c). The device has a floating base, with the intention of providing optical bias. An optically biased device eliminates the parasitic base capacitance that an electrical base contact would necessarily include.

### Transistor Action

**Figure 2:**
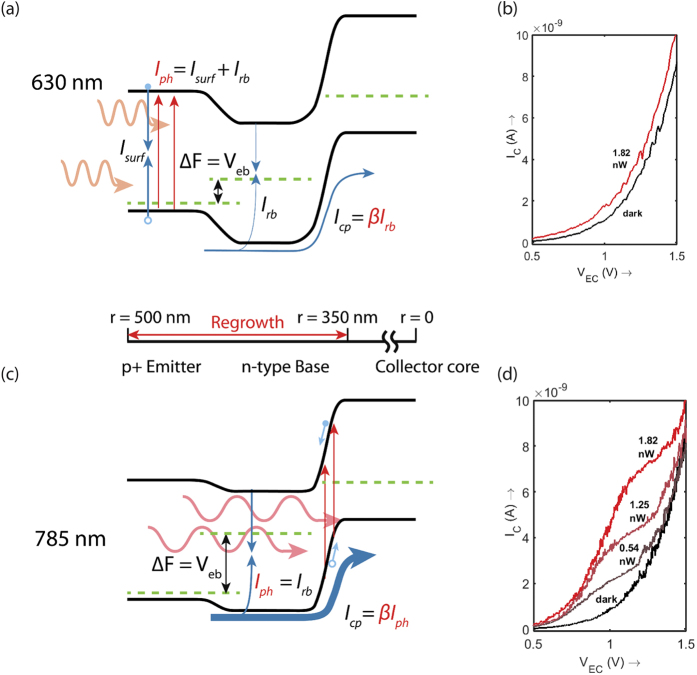
Gain mechanism. The phototransistor gain is illustrated in terms of performance at two different illumination wavelengths for a typical device. Under 630 nm illumination (**a**), the carriers are absorbed very close to the surface since the absorption length is small. This ends up as a surface current and very little excess minority carrier concentration is created at the emitter-base junction. (**c**) On the other hand, at 780 nm illumination, a great fraction of the photons penetrate deeper into the device and the resulting electrons and holes are collected by the reverse biased collector-base depletion. This leads to an effective base current *I*_*rb*_ which is equal to the diode photocurrent *I*_*ph*_. Due to the regrowth-enabled low diode dark current at the emitter-base junction, a large Fermi level split is created. This photovoltage leads to a sufficiently large forward bias on the emitter-base junction in the case of the 785 nm illumination. The photocurrent *I*_*ph*_, which is equal to the base recombination current *I*_*rb*_ is amplified since it turns on a much larger collector hole current *I*_*cp*_. I-V characteristics for a device under (**b**) 630 nm and (**d**) 780 nm illumination shows the more than order of magnitude difference made by the current gain.

Gain in the homojunction bipolar junction transistor (BJT) is obtained when a small base current can reduce the barrier to current flow for a large current from the emitter to the collector^50^,^51^. Considering a p-n-p transistor with heavily doped emitter, a large hole current is injected into the n-doped base when the energy barrier is lowered. A small fraction of these holes recombine with the electron current supplied at the base terminal, but the base length is designed to be short so that a majority of these holes diffuse to the base-collector junction where they are swept by the high reverse bias electric field into the collector (see Fig. 2a,c). The current gain is then the ratio of the collector hole current divided by the significantly smaller portion of the hole current that recombines with the electron current at the base, and can be described using a charge control model as the ratio of two Gummel numbers (further described in Supplementary section II). From this recombination picture, it is clear that for a high gain, it is imperative to have low minority carrier recombination rate in the base – or equivalently a low base dark current.

Exactly the same mechanism is also valid for photogenerated carriers in the device. As described pictorially in Fig. 2a,c, photons generate electron-hole pairs in the absorber – and the electrons lower the energy barrier for the diffusion of holes into the base, a situation similar to the generation of excess carriers in the forward biased base-emitter junction. The quasi Fermi level split(Δ*F*), or equivalently base emitter forward bias voltage(*V*_*be*_) due to excess injected carriers is determined by the optical carrier injection and the electron dark current component at the base-emitter junction:


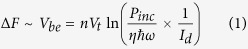


where n is the ideality factor of the diode current, *V*_*t*_ is the thermal voltage of 25 mV at room temperature, *P*_*inc*_ is the incident optical power, 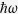 is the photon energy −1.58 eV in our case, *η* the external quantum efficiency and *I*_*d*_ the electron component of the diode dark current. Based on eq. (1), it is clear that it is critical to obtain a very low dark current to attain a large *V*_*be*_ and thus high gain. Hence, having a high quality, defect free p-n-p junction, electically isolated from the substrate is essential. Here, we demonstrate, for the first time, a pristine quality p-n-p junction regrown entirely on an as-grown p-InP nanopillar. As a control device, a continuous p-n-p junction was also grown without regrowth. As can be seen in Supplementary Fig. S1f, the regrown nanopillar photoBJT has a 4 orders of magnitude lower dark current than the shunted junction. Since the shot noise current is proportional to 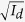, the low dark current also leads to a better sensitivity for the device.

The device operation can further be examined from the wavelength dependent light response Fig. 2. The illumination has been performed at an angle of 30 degrees to the substrate. Light was incident with a single mode fiber and forms a broad Gaussian intensity distribution at the device substrate (further described in Supplementary Fig. S2). The input number of photons is determined purely from this distribution without accounting for the absorption efficiency of the pillar. At 660 nm pump, the light response is poor since the absorption occurs superficially and the electrons recombine at the surface rather than being injected into the base. With 785 nm illumination, the photons penetrate further into the device and lead to carrier generation in the base and collector regions. The electrons lower the barrier to hole current flow, resulting in transistor action and observable gain. The difference in spatial absorption can also be appreciated from the fact that the bandgap for Wurtzite-phase InP at 870 nm, is about 55 nm lower in wavelength compared to Zinc Blende-phase InP, which makes 785 nm close enough to the band edge for the photons to penetrate significantly deeper into the device than the 660 nm case. The 785 nm wavelength was also chosen for characterizing the dynamic response of the device.

### Measurement Results and High Speed Operation

**Figure 3:**
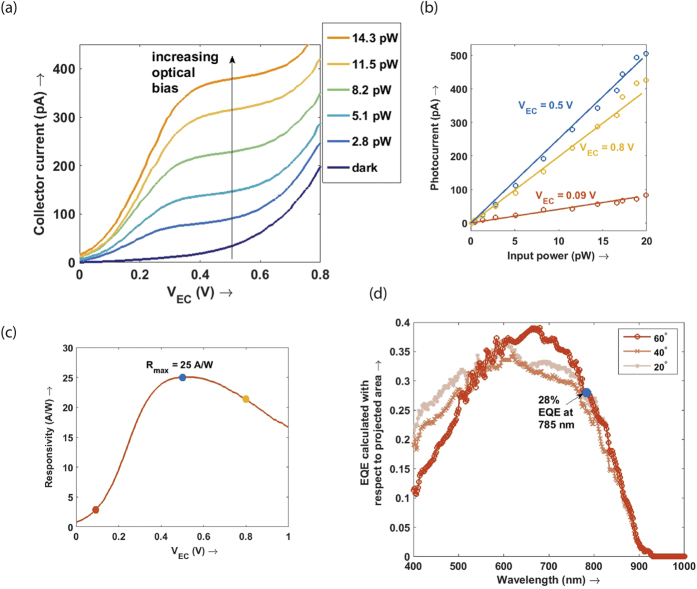
Responsivity. The responsivity for an exemplary single pillar device was measured using illumination at an angle of 60 degrees to the growth axis with a calibrated Gaussian-beam source from a single mode fiber with low numerical aperture. (**a**) The pump dependent response down to 1 pW of optical power was measured. The dark current at 0.5 V bias is as low as 33 pA due to the regrown device structure. (**b**) The photocurrent versus input power shows a linear response, with bias dependent responsivity. At a low collector bias, the device is in saturation mode and the emitter-base junction is not sufficiently forward biased for gain. The optimum bias was 0.5 V, which makes operation at CMOS line voltage possible. On further increasing bias, device punch through leads to a reduction in response. (**c**) A plot of the responsivity vs. bias shows that a peak optical response of 25 A/W can be reached for this device at a 0.5 V bias even at a very low optical power less than 20 pW. (**d**) In order to obtain the current gain or *β* of the transistor, the absorption of the device without current amplification was measured. This is defined as the external quantum efficiency (EQE) as it is a ratio of unamplified electrons out to incident photons. This was measured using a p-i-n junction instead of a p-n-p device, as described in ref. 48 – using similar device dimensions at the sub-micron scale and active region in the regrown part. The EQE was measured to be 28% at an illumination angle θ of 60 degrees to the growth axis (30 degrees to the horizontal) and at a wavelength of 785 nm. The relatively high EQE and angle insensitivity for the nanopillar device is attributed to a dielectric antenna absorption enhancement effect as well as the gold back reflection; coupled with a high current gain this ensures a high optical responsivity.

Figure 3a shows the direct-current (DC) current-voltage (I-V) behavior of an exemplary nanopillar photoBJT from a growth run with optimized doping levels and thicknesses as discussed in the device fabrication section. The total incident power on the device was calculated from measured device dimensions and the expected Gaussian distribution of the optical power from the single mode fiber. Without illumination, the device shows a very low 33 pA of collector current for much of the collector-emitter bias since the p-n junctions inside are reverse biased. On further increasing the collector bias, the current increases due to base width modulation – a common tradeoff in designing narrow base homojunction bipolar transistors^52^.

In forward active mode, the optically biased nanopillar photoBJT shows linear photo response with responsivity approaching 25 A/W, or a gain of 39.5, including the effect of the imperfect external quantum efficiency (EQE) of the device. Figure 3b shows a linear photo response from the nanopillar photoBJT when it is biased with 0.5 V collector bias and observed down to the pW light level. No electric bias was provided to the base junction – a low quiescent optical bias was sufficient to turn the device on, attesting to the quality of the base-emitter junction in the regrowth. A gain of 39.5 for this exemplary device corresponds to a sensitivity improvement of 16.0 dB – which is tremendous given the small noise figure of phototransistors^41^. Such linear and sensitive response is highly desirable in bringing low energy optical interconnects to silicon electronics. Figure 3c shows the responsivity vs. collector bias characteristic in order to highlight key regimes of operation of the device. The responsivity of the device increases with collector bias under saturation regime (between 0 to 0.4 V collector bias), and eventually peaks at 25 A/W at 0.5 V collector bias in forward active mode. As the collector bias continues to increase, responsivity dips. The reduction in responsivity is likely caused by the Early effect observed in the device – equivalently expressed in terms of base width reduction. We also observed base punch-through above 1.0 V collector bias. In order to obtain the true gain, it is necessary to estimate the external quantum efficiency of a device without any transistor gain. This was obtained by measuring the response of a p-i-n junction nanopillar device with the same dimensions and doping levels.

**Figure 4:**
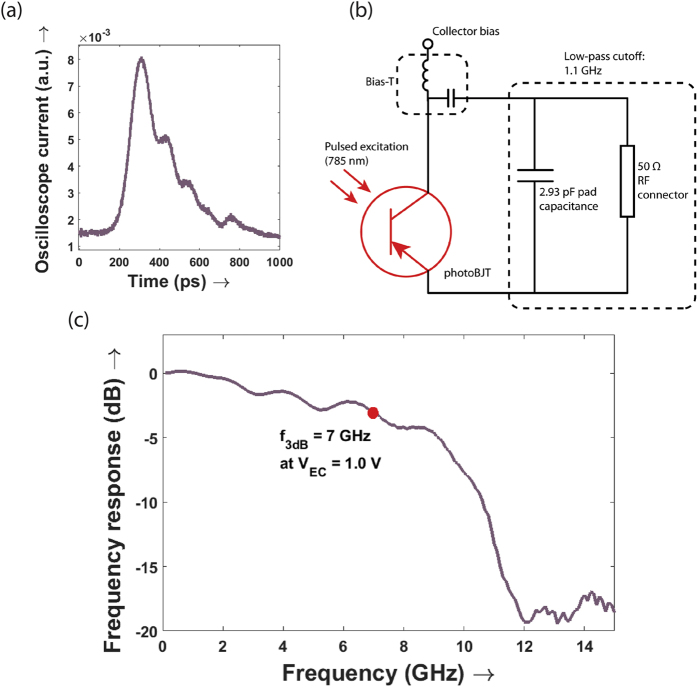
High speed operation. (**a**) The temporal response of the photo-BJT to 100 fs laser pulses at 785 nm wavelength was measured using a high speed oscilloscope. The devices were electrically contacted with 100 μm sized pads to an RF probe. While the measured full duration at half maximum for the device is about 300 ps; to obtain the true device speed, the capacitive effect of the large contact pads needs to be de-embedded. This was done in accordance with the circuit model of (**b**), where the parasitic capacitance was determined using an S11 measurement on an open control device. The de-embedding procedure is described in further detail in Supplementary section III. (**c**) On correctly de-embedding the low-pass filtering effect of the 2.93 pF capacitance on the device, the 3-dB bandwidth was found to be 7 GHz at a voltage bias of 1 V (see Supplementary section III). At the same voltage bias, the device responsivity was also measured and the electronic transition frequency of the device was determined.

Pulsed response measurements were used to obtain the device high speed response for 785 nm illumination. A tunable mode locked solid state laser with small pulse width of ~100 fs was used to excite the device. The device full duration at half maximum from a time resolved measurement, was roughly 300 picoseconds, which corresponds to a device bandwidth of 1.5 GHz. This is limited by the large pad capacitance of the 100 *μm* size contacts – which can be as high as 2–3 pF. For 50 Ω RF connectors, this leads to a low parasitic RC limited bandwidth of 1.1 GHz (Fig. 4b). This pad capacitance can be reduced by an order of magnitude by simply scaling down the size of the contacts and increasing the insulator thickness. Therefore, in order to obtain the true dynamic response of the device, we have de-embedded the capacitive response of the pads from the frequency response of the device, following the circuit model in Fig. 4b. This was additionally verified with an open test device which was fabricated without a nanopillar but on the same wafer (see methods and Supplementary section III for further details on the S11 de-embedding measurement). Using this procedure, the actual 3 dB bandwidth was found to be 7 GHz (Fig. 4c). The 3 dB bandwidth decreases from 7 GHz to 6 GHz on increasing the bias from 1 to 1.5 V. The collector-base depletion region is expected to widen on increasing voltage bias, thus leading to increased transit time. This indicates that the device speed is limited by carrier drift through the collector-base depletion region. The peak power of the pulsed laser is sufficiently high to ensure that the device response is dominated by transit time rather than capacitive charging delays.

**Figure 5:**
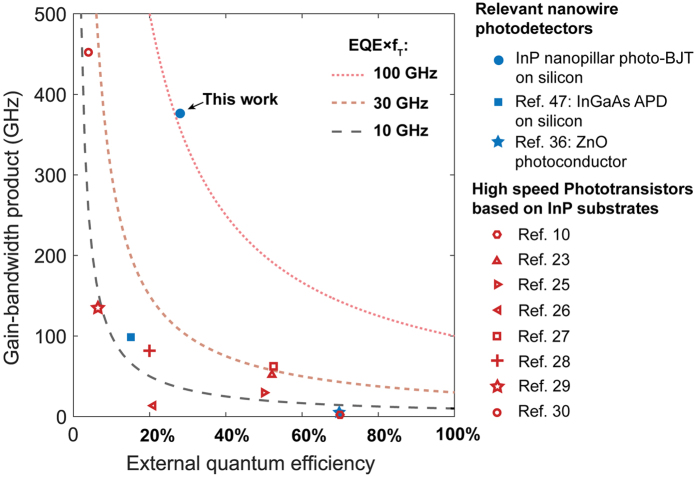
Photodetector performance metrics. For a sensitive high speed detector, it is important to engineer a high gain while operating at high speeds. The Miller capacitance and carrier transit time lead to a combined gain-bandwidth metric (also termed transition frequency, *f*_*T*_) for phototransistors, which can be increased by reducing the device size and engineering a narrow electrical base width^43^. On the other hand, the photon absorption efficiency – which is termed the external quantum efficiency (EQE) - suffers if the device size is too low. Therefore, we have a situation in which conventional transistor scaling leads to extremely high *f*_*T*_ – but with most of the incident photons not being absorbed. Hence, there is a clear need for a metric multiplying *f*_*T*_ with the EQE, which would directly translate into a tangible sensitivity enhancement for light. Isoquants of EQE-*f*_*T*_ product reveal that the reported device has a record performance of 105 GHz EQE-*f*_*T*_ product due to simultaneous compact size and high absorption in a near wavelength-scale InP device. A similar discussion on a ‘multiplied responsivity-bandwidth’ metric has recently emerged for avalanche photodiodes^20^.

The measured responsivity of the 7 GHz high speed device was 9.5 A/W at the same voltage bias, equivalent to an optical gain (electrons out to photons in) of 15. Since this includes the degradation due to non-ideal external quantum efficiency (Fig. 3d), the responsivity translates directly to a sensitivity enhancement of 9.5 dB. To obtain the *β* or transistor current gain of the high speed device, we fabricated a p-i-n junction structure with the same dimensions and measured the external quantum efficiency as a function of wavelength and illumination angle (Fig. 3d). At 785 nm, the measured EQE is 28%, leading to an electronic current gain estimate of 53.6 for a 9.5 A/W responsivity device. The electrical transition frequency (*f*_*T*_) is therefore estimated to be 375 GHz given the measured gain and 3 dB frequency. This approaches the Terahertz scale frequencies of highly scaled heterojunction bipolar transistors fabricated from high quality InP-based systems^53^. This device has the highest gain-bandwidth product accounting for intrinsic photoresponse (or equivalently, EQE-*f*_*T*_ product) in the published literature for InP-based phototransistors, even though higher electronic *f*_*T*_ has been obtained with a drastic penalty on the light absorption (see Fig. 5 and Supplementary Table 3). A high gain bandwidth product also necessitates a sufficiently high quiescent bias. Here, the bias is provided optically – which has the advantage of eliminating any base capacitance that an electrical bias would necessarily have.

The high light absorption efficiency for such a compact structure has been validated with angle resolved external quantum efficiency measurements as well as 3D finite difference time domain simulations^48^. The enhanced absorption can be elucidated in terms of the high-index dielectric antenna characteristics of the nanopillar absorber^48^,^54^ combined with the double pass due to the back reflection from the contact metal.

## Discussion

By engineering a suitable doping profile in a compact InP nanopillar device, a bipolar transistor gain mechanism has been utilized for high gain-bandwidth product while maintaining high absorption – a completely novel demonstration for a bottom-up nanowire device, thus validating this technology for high speed multi-Gigahertz optical receivers integrated on chip. This has been accomplished through a careful combination of bottom up nanopillar growth/regrowth as well as top-down device processing. With a highly scaled geometry free of excessive surface recombination, the nanopillar bipolar junction phototransistor is able to reach a current gain of 53.6 at a low bias voltage of 1 V and show a high bandwidth of 7 GHz while maintaining a good intrinsic photoresponse.

**Figure 6:**
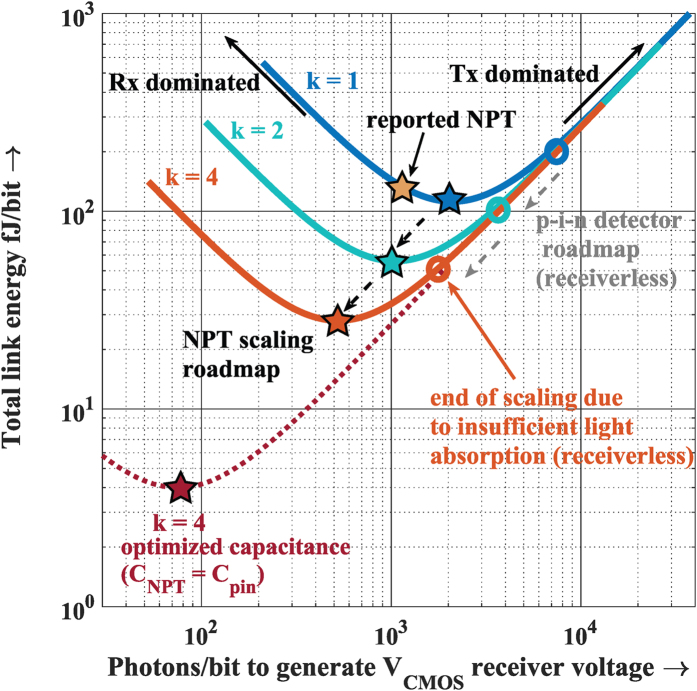
Link energy consumption for nanopillar phototransistor receiver. The total link energy versus photons/bit (or equivalently optical power) absorbed by the photodetector for generating a 0.6 V output voltage. With an implicit parameter of the current gain *β* for this plot, the link energy consists of a sum of the receiver and transmitter energy consumption. The photons per bit are inversely proportional to *β* and directly proportional to *C*_*tot*_, the device capacitance. Thus, we see that despite a larger capacitance in the nanopillar phototransistor, the link can have lower minimum energy consumption compared to the receiverless case (56 fJ/bit vs. 100 fJ/bit, k = 2 node). Three different curves have been plotted for the three different scaling nodes (see Tables 1 and 2, Supplementary section V). It can be seen that the receiverless link is dominated by transmitter power at all three nodes^2^. With optimization, the capacitance of the nanopillar phototransistor can approach that of the receiverless detector^3^,^60^ at the same scaling node, leading to an even more compelling energy reduction (dashed curve). It is expected that conventional scaling for photodetectors will plateau at the k = 4 scaling node, beyond which the absorption efficiency will be impractically low, and energy per bit can no longer continue to decrease. However, the nanopillar phototransistor allows the energy per bit to decrease by an order of magnitude beyond this, even at the same dimensional scaling node. A similar analysis for energy/bit was also reported in ref. 12.

On a detailed analysis of energy consumption of the entire link based on the roadmap outlined in Manipatruni *et al*.^2^, it has been found that the inclusion of gain in the photoreceiver allows for a significant reduction in transmitter energy, which dominates the link power consumption. The link energy/bit has been calculated with the requirement that the photons absorbed by the photodetector lead to a CMOS-level voltage swing at the output of the photoreceiver. It has been found that the integrated gain leads to a significant reduction of the photons/bit and hence reduced total link energy consumption (Fig. 6). This is in spite of the excess power expenditure at the nanopillar phototransistor compared to the receiverless case (see Supplementary Materials Section V, Tables 1 and 2 for further details).

The importance of reducing total capacitance for a highly sensitive photoreceiver has been emphasized and also addressed using an extremely compact InP device with high absorption. Further improvements in device characteristics are expected with a better electrical contact geometry to mitigate pad capacitances. Additionally, the incorporation of a thin InGaAs base in a highly scaled nanopillar heterojunction bipolar phototransistor structure is expected to lead to near-THz cutoff frequency, with less than 10 fJ/bit total link energy (Supplementary Table 1). The core-shell growth of high quality InGaAs layers in InP nanopillars has already been demonstrated^46^,^55^,^56^ and will be used to further pursue the implementation of ultrahigh performance photoreceivers for CMOS compatible photonic integrated circuits.

## Methods

### Nanopillar Device Fabrication

The growth was accomplished on silicon substrates via metal organic chemical vapor deposition (MOCVD) at a CMOS compatible temperature of 450 °C, for a low growth time of less than 30 minutes^57^,^58^,^59^. Within a narrow growth window, tuning the temperature and mole fractions of the trimethylindium and tertiarybutylphosphine precursors to 4.73 × 10^−6^ and 5.94 × 10^−4^ respectively leads to the spontaneous nucleation of InP nanopillars on the chemically roughened Si-111 oriented substrate. The nanopillar scales in size with increasing growth time, beyond even a micron for 15–20 minutes of growth. Sharp electrical junctions are grown using *in-situ* doping during epitaxial core-shell growth. Zn dopant precursor (diethylzinc) was used for the p-type doping and Te doping (diethyltellurium) for the n-type base.

First, the low p-doped core nanopillar is grown to a thickness of 800 nm. The sample is then removed from the growth chamber and covered with silicon dioxide and then amorphous silicon using plasma enhanced chemical vapor deposition (PECVD) at a low substrate temperature of 250 °C. The outer sleeve is then etched back from the upper half of the pillar using a photoresist mask. The oxide+a-Si sleeve on the lower half of the pillar serves to allow regrowth only on the top exposed section. The sample is then placed back in the MOCVD chamber and sequentially regrown with 25 nm of p-doped InP, 75 nm of n-doped InP for the base and ultimately 50 nm of p-doped InP for the emitter.

Two electrodes are defined for high speed ground and signal contacts to an individual nanopillar photoBJT^39^, with electrode size of 100 *μm* × 100 *μm* and oxide isolation to the substrate with a 200 nm thick silicon dioxide layer (see device SEM and schematic cross section, Fig. 1a,b). Angled electron beam evaporation of titanium/gold contacts is performed, in order to allow for angled illumination of the device.

### Optical characterization

All characterization was performed under ambient, room temperature conditions. Illumination was provided with a single mode fiber angled at 30 degrees from the horizontal. The distance from the fiber tip to the substrate was measured to be 2.16 mm. This leads to a Gaussian distribution with a spot radius of 220 *μm* at the substrate, which is large enough to assume a constant intensity distribution across the micron size device. The device was placed at the peak intensity position, and the response was estimated by calculating the device cross section from scanning electron microscope images of the device dimensions.

### Dynamic response

A high speed bias-T was used to provide a DC bias of 1 V and also route the RF signal and measure it on a triggered high speed oscilloscope. An HP4145B semiconductor parameter analyzer was used to apply the collector bias, while the high speed response was routed to a Keysight 86100A DCA wide-bandwidth oscilloscope. This was triggered with a signal from a high speed photodetector which was simultaneously illuminated with the femtosecond pulsed laser.

The de-embedding measurement was performed with an S11 characterization of the RF reflection for an open test device on the same wafer as the fabricated nanopillar photoBJT devices. The open test device had the same contact pad size as the high speed phototransistor (100 *μm* ground and signal pads). A reference load (50 Ohm), short and open test wafer (Cascade 103-726) was used to calibrate the RF cables and probes to ensure the accuracy of the reflection measurement. The measurement was then performed up to an RF frequency of 20 GHz with a Keysight E8361A PNA Network Analyzer.

## Additional Information

**How to cite this article**: Ko, W. S. *et al*. Ultrahigh Responsivity-Bandwidth Product in a Compact InP Nanopillar Phototransistor Directly Grown on Silicon. *Sci. Rep.* **6**, 33368; doi: 10.1038/srep33368 (2016).

## Supplementary Material

Supplementary Information
