# Supplementary material for: Ultrahigh Responsivity-Bandwidth Product in a Compact InP Nanopillar Phototransistor Directly Grown on Silicon
Source: Sci Rep. 2016 Sep 23;6:33368. doi: 10.1038/srep33368 (PMC5034248; doi:10.1038/srep33368)
Supplement: Supplementary Information [file srep33368-s1.pdf]

Supporting information:

Ultrahigh Responsivity-Bandwidth Product in a Compact InP Nanopillar Phototransistor Directly Grown on Silicon

Wai Son Ko*, Indrasen Bhattacharya*, Thai Tran D. Truong, Kar Wei Ng,

Stephen Gerke, Connie Chang-Hasnain

Department of Electrical Engineering and Computer Sciences, University of California at Berkeley, Berkeley, California 94720, United States of America

*equal contribution

I. Device fabrication process and importance of regrowth..

II. Current gain determination..

III. S11 de-embedding measurement...

IV. Gain bandwidth product discussion ....

V. Total link energy benefit and scaling considerations ....

VI. Prospects for silicon photonics integration……………………………………………………

VII. High speed photodetector metrics ....

I. Device fabrication process and importance of regrowth

A two-step growth method was used to synthesize the nanopillars. The p-core collector region was grown first, followed by regrowth selectively on the upper region of the nanopillar. The core of the phototransistor was grown directly on silicon and p-doped in-situ (Fig. S1a). Using a calibration of the core-shell growth rate from a timed growth study (Ref. S2) we determine that 25 nm zinc doped (p-emitter), 75 nm tellurium doped (n-base) and 100 nm zinc doped (p-collector) layers were respectively regrown onto the upper portion of the nanopillars at 450°C. The emitter was doped slightly higher than the collector. The base doping was approximately $1 \times 10^{18}\ cm^{-3}$, as determined by a Burstein-Moss shift in photoluminescence. The doping levels were kept high for the high speed devices in order to keep junction depletion lengths small and minimize transit times.

The device was fabricated to make sure contact was achieved with an individual pillar, and that the top contact is insulated from the silicon substrate. A silicon dioxide sleeve (green in Fig. S1d) was defined with plasma-enhanced chemical vapor deposition at 250°C. The height of the oxide sleeve was controlled with a timed oxygen plasma etch of a photoresist mask, followed by a buffered hydrofluoric acid etch. The oxide sleeve serves to electrically isolate the top contact from the ground. In addition, this timed plasma is also used to control the emitter contact length to 1 micron. The small size of the junction helps in reducing device capacitance.

Finally, the contacts were defined using metal evaporation and lift-off. First, the ground contact was defined by selectively etching through the oxide using a photoresist etch mask and a timed buffered hydrofluoric acid etch. Then, using a separate mask, the ground and top contacts were simultaneously patterned for metal evaporation, using e-beam lithography. The contact layers were deposited using angled electron-beam evaporation under high vacuum: first a 10 nm titanium layer, and then 150 nm of gold. The angled e-beam process creates a shadow on the substrate, as can be seen in the scanning electron microscope image of Fig. 1b in the manuscript. The exposed (non-metallized) half of the device permits angled light illumination, which was performed at 30 degrees angle to the substrate (see Fig. S2).

A control device was also fabricated using direct growth of all layers. As can be seen in Fig. S1e, this leads to the device being shunted to the substrate. Measurement of the dark i-v characteristic (Fig 1f) of the devices shows that the regrown device has 3-4 orders of magnitude lower leakage. This is the key factor in allowing the regrown devices to show response to extremely low levels of light.

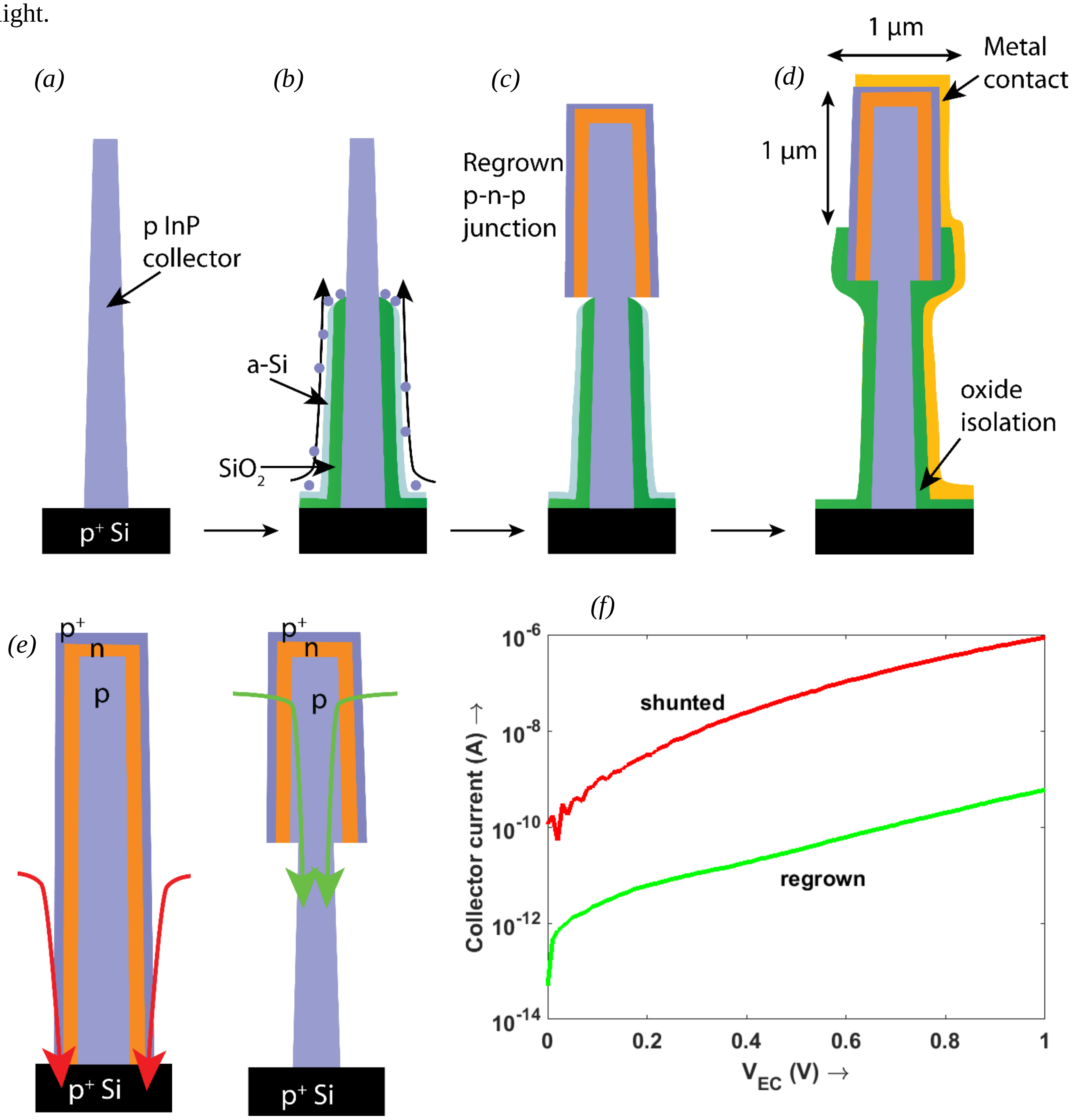

Fig. S1: a – d: condensed schematic of the growth and fabrication process for the nanopillar phototransistor (a: original growth, b,c: regrowth, d: final device structure, e: comparison of current flow paths for regrown vs. non-regrown device, f: dark i-v characteristics for the same

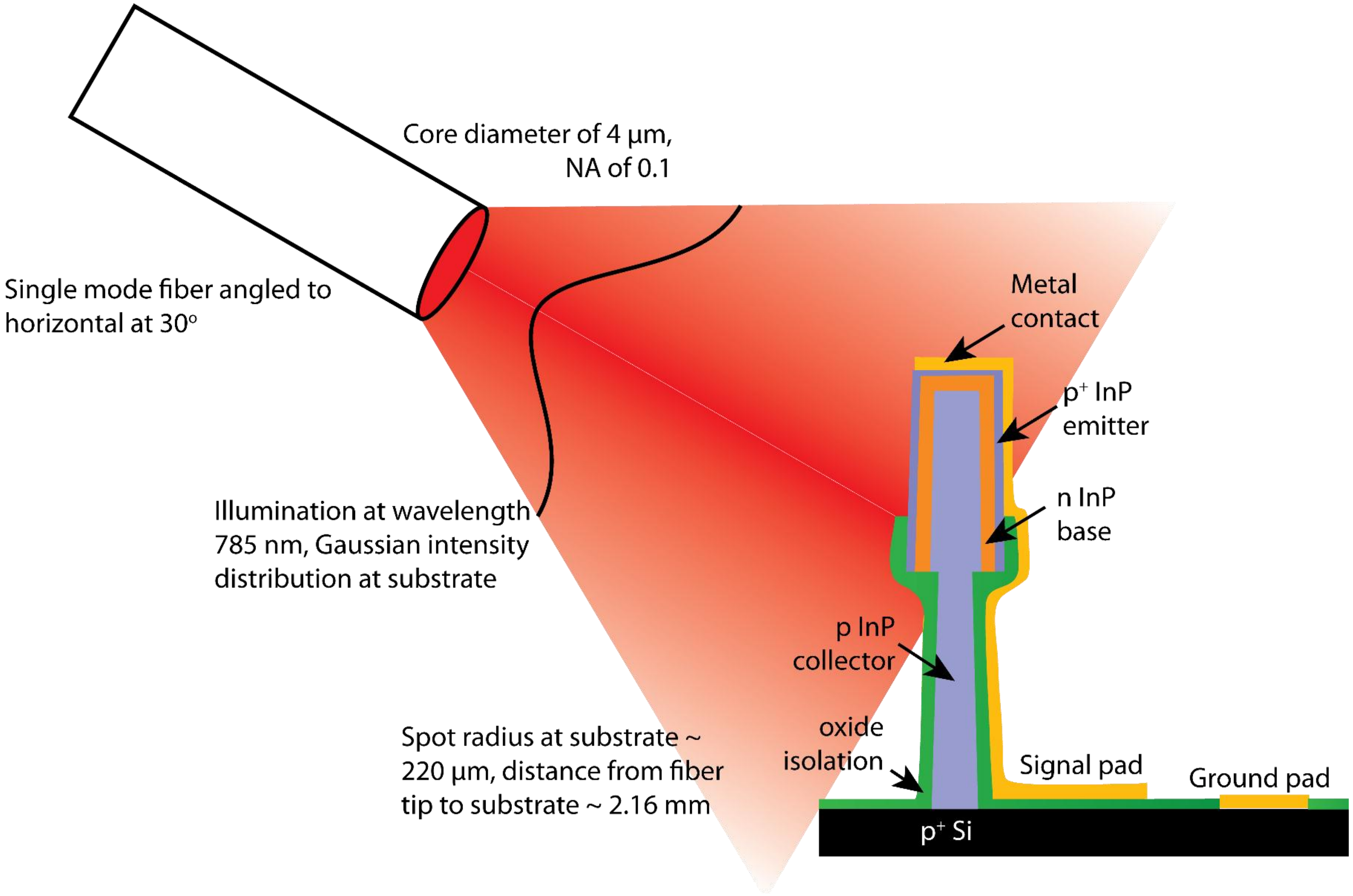

Fig. S2: The optical response was measured with effectively collimated illumination from a single mode fiber source with a low numerical aperture and spot size on substrate which was two orders of magnitude larger than the pillar. The fiber was angled to a configuration for which the absorption efficiency had been measured, and the spatial power distribution is well described as Gaussian since the fiber is single mode at 785 nm.

II. Current gain determination

The optoelectronic response of the device was measured using calibrated illumination from a 785 nm laser source shone on the device in an angled geometry as depicted in figure 2a. The fiber-coupled source has a low numerical aperture of 0.1 and the illumination spot size is several orders of magnitude larger than the pillar, so the illumination can be assumed to be spatially invariant across the pillar. The position of the pillar was optimized to the peak of the Gaussian

illumination spot. The responsivity was determined by calculating the ratio of the photocurrent in amperes to the absolute optical power incident on the projected cross section of the pillar. The responsivity was determined to be 9.5 A/W for an exemplary high speed device, which corresponds to an optical to electronic gain of 15 for 785 nm photons. In order to determine the true electronic gain or β, the absorption efficiency was experimentally determined in the same geometry for various angles and resolved against wavelengths, as illustrated in fig. 2d in the manuscript. A Xenon arc lamp source, filtered through a monochromator was used for the measurement. The absorption efficiency of photons, which we term the external quantum efficiency to be consistent with published literature, is as high as 28% for such a compact device geometry.

The electronic gain is then as high as 53.6 (obtained by dividing the optical gain by the absorption efficiency). In a homojunction bipolar junction transistor, the current gain is determined by a ratio of the two Gummel numbers for the emitter and base currents (see for instance, Ref. S3, chapter 8):

$$\beta = \frac{G_E}{G_B} = \frac{W_E N_E D_B}{W_B N_B D_E}$$

where $W_{E(B)}$ is the undepleted electrical width of the emitter (base), $N_{E(B)}$ is the emitter (base) doping concentration and $D_{B(E)}$ is the base (emitter) minority carrier diffusion coefficient. Therefore, we have kept the electrical base width low and emitter doping high in order to achieve a high current gain. Current gain on the order of 100 is routinely achievable with BJTs and even higher with heterojunction bipolar transistors.

III. S11 de-embedding measurement

As shown in Fig. 4b of the manuscript, the parasitic pad capacitance has the effect of low pass filtering the high speed current signal from the phototransistor. In order to determine the true response, open devices with the same pad dimensions of $100\ \mu m \times 100\ \mu m$ and same oxide thickness were fabricated adjacent to the nanopillar devices. An S11 reflection measurement was

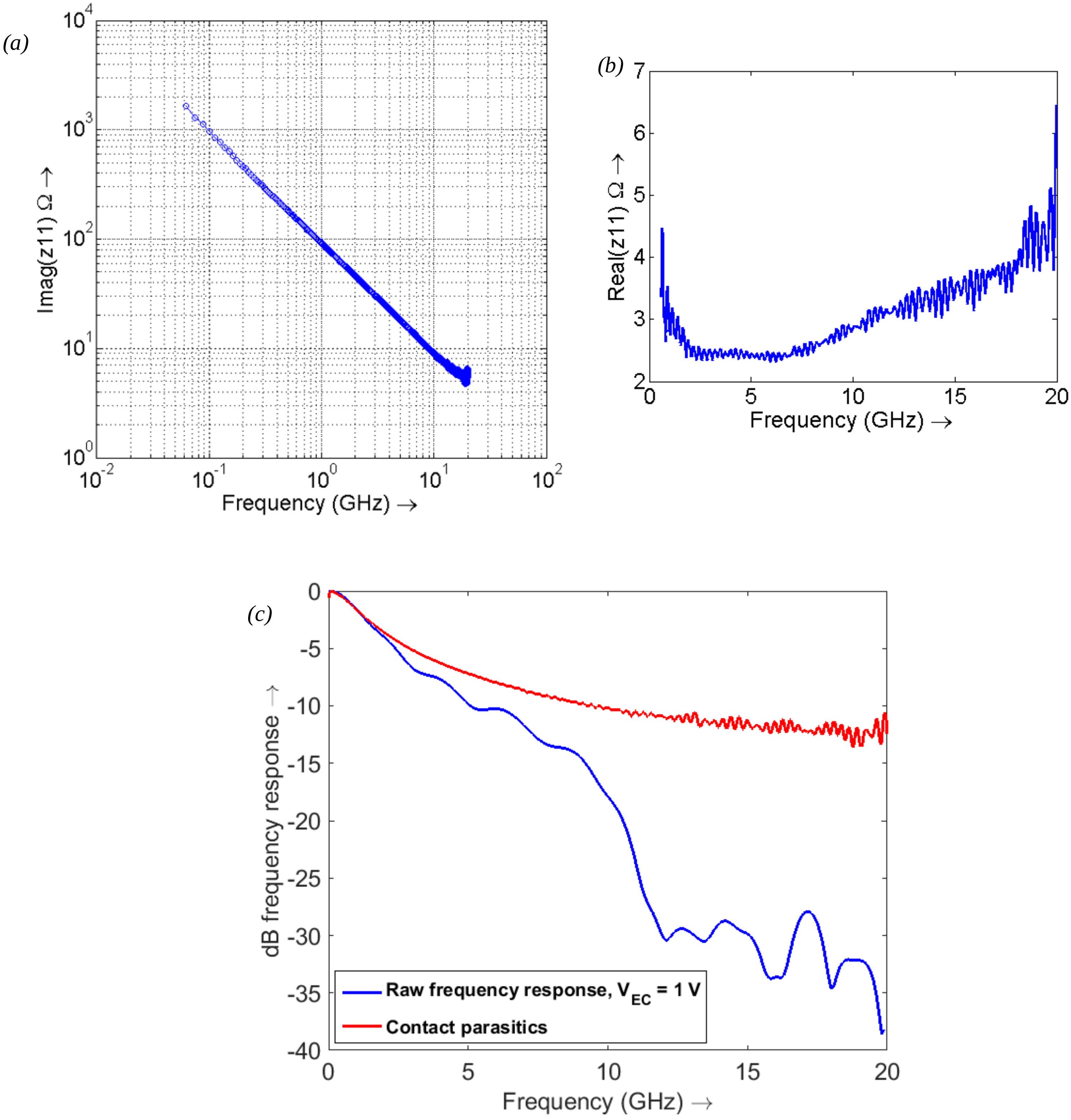

Fig. S3: (a) The imaginary part of the impedance of the open pad shows a capacitive response corresponding to 2.93 pF, due to the somewhat large size of the pads and thin oxide. At 10 GHz, the impedance is as low as 10 Ω, meaning that the high speed response is shunted. (b) The small real part of the impedance corresponds to the series resistance to the contact. (c) Plotting the parasitic transfer function and the raw frequency response of the device shows that the response is determined by the parasitics at low frequency, and de-embedding actually leads to a high 3 dB cut-off frequency of 7 GHz as shown in the manuscript.

used to determine the S-parameters of the open device. Agilent E8361 PNA network analyser was used to perform the measurement. The S parameters were then converted to corresponding impedances, or Z parameters. The plot in Fig. S3a shows the absolute value of the imaginary part of the impedance of the open device as a function of frequency. The first order roll off clearly indicates a capacitive transfer function: $Z = \frac{1}{2\pi jfC}$, which is fitted to a capacitance of 2.93 pF. There is also a small real part to the pad impedance from the series resistance of the gold contact (2-5 Ω). Combined with the 50 Ω standardized RF cable, this leads to a low-pass transfer function corresponding to a 3dB cut-off frequency of ~1 GHz, which completely overwhelms the true high speed response (as seen in Fig.S3 c). On dividing the fourier transform of the pulsed temporal response by the parasitic transfer function we obtain the true 3 dB frequency of the device. The capacitance can be reduced by using smaller pads and a thicker oxide insulator.

IV. Gain-bandwidth product discussion

Having determined the low frequency current gain of 53.6 and the true 3 dB bandwidth of 7 GHz for the same phototransistor device, operating at 1 V of voltage bias – we calculate the electronic gain-bandwidth product or transition frequency f_T to be 375 GHz. We briefly discuss the factors that determine f_T for the reported device.

The temporal delays that determine f_T consist of the RC limit due to the charging of the device internal capacitances, the base transit time and the collector depletion layer transit time:

$$Gain \times BW = f_T$$
$$= \frac{1}{2\pi} \times \left(\frac{V_t C_{tot}}{I_c} + \frac{W^2}{\eta D_B} + \frac{x_{dep}^c}{2v_{sat}} \right)^{-1} \quad (2)$$

where I_c is the collector bias current, V_t is the thermal voltage of 25 mV, C_{tot} consists of the parallel combination of base emitter and base collector capacitances, W is the undepleted base width, D_B is the hole diffusion coefficient in the base for our hole injection device, η is a

dimensionless number describing the base transit time, that can be increased by engineering a built in field, x_{dep}^{c} is the collector depletion region width and v_{sat} is the hole saturation velocity (see Ref. S4, section 3.3.1 'Cutoff frequency'). We would like to note that carriers do not need to be generated in the base: if the electrons can diffuse to the base-collector junction faster than the 3 dB frequency, or equivalently the data rate, then they can be amplified by fast transistor action. This is a much less stringent requirement than the f_T for the amplified carriers since it is not subject to the Miller effect.

The capacitance limited charging time is dependent on the collector current through the RC-time: CV_t/I_c where C is the total device capacitance, I_c is the collector current and V_t is the thermal voltage. The capacitance is estimated using a co-axial capacitor model:

$$C = \frac{2\pi\epsilon_o\epsilon_{InP}L}{\ln\left(\frac{r_{out}}{r_{in}}\right)}$$

where the depletion region extends from a radius of r_{in} to r_{out}. In our case, the junction length L is determined by the regrowth oxide mask to be 1 μm. The depletion regions are rather thin at 40-50 nm, due to the high doping. The average radius of the junction is estimated to be 450 nm for the C-B junction and 500 nm for the E-B junction (based on growth time as in Ren *et al.*, Ref. 45 in the main text). We estimate the emitter base junction capacitance to be 7.6 fF and the collector base junction capacitance to be 6.6 fF. Such low capacitance values can be attained in spite of the thin depletion regions due to the compact size of the device. The forward-biased emitter-base junction also has capacitance due to minority carrier charge storage in the base, which has been accounted for in the transit time calculation.

The f_T increases by increasing the collector current until the transit time limit sets in – which provides an upper limit on the device gain bandwidth product. We have approached the transit limited bandwidth by using a pulsed measurement in which the peak current can be sufficiently high. The average optical power used for the pulsed measurement was $P_{abs} = 150$ nW absorbed by the pillar at $\lambda = 785$ nm wavelength. Given the laser pulse period T of 12.5 μs, the typical minority carrier lifetime in the doped semiconductor τ to be 1 ns (Ref. S5), the fundamental

charge q, and the base for the natural logarithm e, we estimate the average quiescent base bias current in each pulse:

$$I_b = \frac{1}{e} \times q \times \frac{P_{abs}T}{\frac{hc}{\lambda}} \times \frac{1}{\tau} \sim 430\ \mu A \tag{3}$$

The reported transition frequency of 375 GHz is well within the upper limit imposed by the capacitive charging limit ($= 1/2\pi \times \beta I_{dc}/CV_t$) for the above base current and junction capacitances. Thus, using the pulsed measurement, we have effectively measured the transit time limit to the gain bandwidth product for our device geometry.

V. Total link energy benefit and scaling considerations

The total link energy is calculated based on the somewhat stringent requirement that the photons absorbed at the photodetector end of the optical link should be sufficient in number to result in the total receiver capacitance being charged up to a CMOS level voltage swing:

$$V_{CMOS} = \frac{Q}{C_{tot}} = q\left(\frac{\boldsymbol{P_{abs}}}{\hbar\omega \times f_{data}}\right) \times \frac{1}{C_{tot}} \tag{4}$$

where P_{abs} is the optical power absorbed, $\hbar\omega$ is the photon energy, f_{data} is the data rate in the link and C_{tot} is the total receiver capacitance. This is for the case of an amplifier-less photodetector with no additional gain – such as a p-i-n photodiode (also termed 'receiverless' detector). However, in the case of a photoreceiver with integrated gain, such as the phototransistor, the optical power requirement to reach the same voltage swing is lowered significantly due to the in-built amplification. There is some energy cost in the amplification, but this is offset by the reduction in transmitter power, which usually dominates the total link energy consumption. In what follows, we will justify that the total link energy is significantly less for a link with a phototransistor compared to a conventional p-i-n detector.

For consistency with *Manipatruni et al.* (Ref. 2 in the main text), we use 0.6 V as the required voltage swing. From the same reference, the source energy/bit is then derived by accounting for

the link losses (coupling, insertion and other optical losses), as well as the detector and laser efficiencies:

$$E_{src} = \frac{\boldsymbol{P_{abs}}}{f_{data}} \times \frac{1}{\eta_D \eta_L \eta_M \eta_C} \times 10^{\alpha L} \times \frac{1}{\boldsymbol{\beta}}$$
$$= V_{CMOS} \boldsymbol{C_{tot}} \times \frac{\hbar\omega}{q} \times \frac{1}{\eta_D \eta_L \eta_M \eta_C} \times 10^{\alpha L} \times \frac{1}{\boldsymbol{\beta}}$$
$$= E_{src}(Rxless) \times \frac{\boldsymbol{C_{tot}}}{C_o} \times \frac{1}{\boldsymbol{\beta}} \quad (5)$$

where the notation in the first line is based on the calculation in *Manipatruni et al.*, but modified to include the reduction in transmitter power due to photo-BJT current gain β. In addition, the total capacitance of the phototransistor C_{tot} may be different from that of the roadmap device C_o(= 1 fF). Finally, this leads to the third line, where the transmitter energy is related to the receiverless case, while accounting for the current gain and capacitance of the receiver. The estimated receiverless source energy (not including wavelength tuning) in *Manipatruni et al.* is 50 fJ/bit under favourable assumptions for the link losses and quantum efficiencies (total 3 dB, including detector quantum efficiency). In conventional optical links however, a link margin as large as 5-10 dB is included, beyond what is dictated by the link losses. Therefore, we make the assumption that an additional 3 dB of margin or unaccounted loss has to be included, and proceed with 100 fJ/bit purely for the source energy consumption.

The receiver part of the energy consumption is estimated as follows. In the amplifier-less link, the p-i-n photodiode consumes a relatively negligible amount of energy:

$$E_{Rx}(Rxless) = \frac{1}{2} C_o V_{CMOS} V_d \quad (6)$$

where V_d, the p-i-n junction bias, is assumed to be set to 1 V. For the roadmap of 1 fF for C_o the photodiode capacitance, this leads to an energy consumption of 0.3 fJ/bit. Since the receiver energy is minuscule compared to the transmitter part, it makes sense to integrate gain, even with some energy penalty. We estimate the phototransistor gain assuming the high speed response is

limited by the capacitive charging time rather than the carrier transit time. This has been the case for heterojunction bipolar transistors in the recent past (*Hafez et al.* Ref. 52 in the main text) due to extensive vertical scaling and attendant transit time reduction in such InP-based devices. In that case, to reach a gain-bandwidth product of f_T, the collector DC bias current must be:

$$I_c = 2\pi f_T C_{tot} \times \frac{kT}{q} \tag{7}$$

where kT/q is the thermal voltage of 25 mV and C_{tot} is the total device capacitance.

The energy per bit for the receiver part can then be estimated:

$$E_{Rx} = \frac{I_c V_d}{f_{data}}$$

$$= E_{Rx}(Rxless) \times \boldsymbol{\beta} \times \frac{\boldsymbol{C_{tot}}}{\boldsymbol{C_o}} \times \frac{4\pi\left(\frac{kT}{q}\right)}{V_{CMOS}} \tag{8}$$

$E_{Rx}(Rxless)$ is the previously calculated energy consumption of only the p-i-n photodiode, which is 0.3 fJ/bit. We have substituted the required I_c for a given f_T from (7), and used that f_T/f_{data} is the available gain of β. This leads to the second line in equation (8), where the receiver energy consumption for the phototransistor is related to the receiverless energy consumption by a multiplication factor corresponding to the gain and device capacitance. On the other hand, there is an advantage inherent to the BJT operation mechanism: $\frac{\left(\frac{kT}{q}\right)}{V_{CMOS}}$ that is related to the high transconductance associated with the bipolar gain.

We continue the analysis in order to determine the optimal bias conditions on the receiver phototransistor so that the entire system energy is minimized. We substitute the values of the receiverless energies calculated above based on the roadmap optical link in *Manipatruni et al.* ($E_{Tx}(Rxless) = 100$ fJ/bit and $E_{Rx}(Rxless) = 0.3$ fJ/bit). The current gain β needs to be chosen to minimize the total energy per bit E_{tot}:

$$E_{tot} = E_{Tx} + E_{Rx} = \frac{C_{tot}}{C_o} \times \left(100\ \text{fJ/bit} \times \frac{1}{\boldsymbol{\beta}} + 0.3\ \text{fJ/bit} \times \boldsymbol{\beta} \times \frac{\left(\frac{4\pi kT}{q}\right)}{V_{CMOS}}\right) \tag{9}$$

This formula expresses the idea that the phototransistor gain can be used as an additional degree of freedom to optimize the link performance, by trading off the source power requirement with the amplification energy. This is explicitly graphed in fig. 6 in the main text. The optimal gain β is calculated to from (9) to equal $\sqrt{\frac{E_{Tx}(Rxless)}{E_{Rx}(Rxless)} \times \frac{V_{CMOS}}{4\pi\frac{kT}{q}}} \sim 25$, which is consistent given the significant asymmetry in the receiver and transmitter power consumption in the amplifier-less link. With this optimized gain, the transmitter energy is reduced to about 4 fJ/bit and the receiver energy is increased to 4 fJ/bit – leading to a much reduced total link energy of 8 fJ/bit compared to the amplifier-less value of 100 fJ/bit in the roadmap.

This order of magnitude reduction has been calculated in the case that the total photoreceiver capacitance at the same scaling node is equal to the p-i-n diode capacitance. However, due to narrower junctions, the total capacitance of the NPT reported here is ~14 fF, which is currently somewhat high compared to ~2 fF for optimized germanium p-i-n photodiodes reported in the literature (eg: *Chen et al.*, Ref. 3 in the main text). Despite the higher capacitance of this device, the link energy would be 56 fJ/bit, half that of the receiverless case.

The tables (Table 1 and 2) below summarize the calculated energy per bit and other link parameters based on the above analysis. The blue rows correspond to the current scaling node, where the p-i-n diode capacitance is 2 fF. We estimate that the receiverless link will consume ~200 fJ/bit. On the other hand, the estimated energy consumption of our current device, with sufficient bias to lead to a 375 GHz capacitive charging-limited gain-bandwidth product, is 146 fJ/bit. This can be further reduced to 112 fJ/bit by optimizing the gain. As discussed in the above analysis, the receiver and transmitter consume equal power at the optimal gain.

Additionally, we can also make some dimensional scaling arguments for the photoreceiver performance. The per channel data rate at each scaling node is expected to be determined by system consideration. Given the available device speeds and modulator performance, we specify the current node data rate as 10 Gb/s, and scale to 20 Gb/s for the next node (k=2, cyan) and 40

Gb/s (k=3, orange) – with 40 Gb/s being the final node of the roadmap in *Manipatruni et al.* At every node, the device dimensions scale by a factor of 2 in our analysis, leading to the capacitance correspondingly reducing by that factor. As a conservative estimate, we assume that at every node, the phototransistor capacitance is 7x that of the p-i-n diode. Even with this estimate, the photo-BJT consumes only about half of the energy of the receiverless system at the corresponding node. Further, with improved device design and scaling of the emitter-base junction while maintaining the collector-base junction size at every node, we expect to make the capacitance of the phototransistor approach the capacitance of the p-i-n photodiode at that node. Under this condition, the energy/bit is reduced more than an order of magnitude compared to the receiverless system (Fig. 6, main text).

Table 1: Scaling Projections for Nanopillar Phototransistor

k (scaling node)	Description	Data rate	β	f_T (Electronic Gain-Bandwidth)	Capacitance	Transmitter energy	Receiver energy	Total energy
1	NPT in this paper	7 Gb/s	54	375 GHz	14 fF	26 fJ/bit	120 fJ/bit	146 fJ/bit
1	NPT in this paper with optimized bias to minimize link energy	10 Gb/s (<15 Gb/s)	25	375 GHz	14 fF	56 fJ/bit	56 fJ/bit	112 fJ/bit
2	Optimized bias (min energy)	20 Gb/s (<30 Gb/s)	25	750 GHz	7 fF	28 fJ/bit	28 fJ/bit	56 fJ/bit
4	Optimized bias and capacitance	40 Gb/s	25	1.5 THz	0.5 fF	2 fJ/bit	2 fJ/bit	4 fJ/bit

		(<60 Gb/s)						

We would also like to emphasize that the scaling is performed both ‘vertically’ (epitaxial layer thickness reduction) and ‘laterally’ (area reduction), to be consistent with terminology used in the reported body of work for heterojunction bipolar transistors. Thus, as we scale down, the gain-bandwidth product also increases linearly. In further detail:

$$f_T = \frac{1}{2\pi} \times \frac{1}{(C_{eb}+C_{bc})\frac{1}{\boldsymbol{k}}\times\frac{k_B T}{q}\times\frac{1}{I_c}+\frac{(W/\boldsymbol{k})^2}{\eta D_B}+\frac{(x_{dep}^{c}/\boldsymbol{k})}{2v_{sat}}} \tag{10}$$

where the notation is the same as in equation (5) above, with the understanding that the quantities are referred to a base dimensional node (current device size). The factor *k* is the dimensional scaling factor, with, the same meaning as in Table 1. A factor of *k* scaling is taken to imply simultaneous ‘vertical scaling’ as well as ‘lateral scaling’ by the same factor, so the device aspect ratio is maintained in this scaling analysis. Vertical scaling of the epitaxial layer thicknesses is achieved through growth time reduction, as demonstrated in the core-shell growth mode (*Ren et al.*, ref. 45 in the main text). Lateral scaling is achieved by reducing the active region height by controlling the height of the regrowth oxide sleeve.

Table 2: Scaling Projections for Receiverless Detector

k (scaling node)	Data rate	f_{3dB} (device bandwidth)	Capacitance	Source energy	Receiver energy	Total energy
1 (similar to Ref. 3 and Ref. 60 in main text)	10 Gb/s	45 GHz	2 fF	200 fJ/bit	0.6 fJ/bit	200.6 fJ/bit
2	20 Gb/s	45 GHz	1 fF	100 fJ/bit	0.3 fJ/bit	100.3 fJ/bit
4	40 Gb/s	45 GHz	0.5 fF	50 fJ/bit	0.15 fJ/bit	50.15 fJ/bit

In the case of the waveguide integrated germanium photodetector (Chen *et al.*, Ref. 3 and DeRose *et al.*, Ref. 60 in the main text) dimensional scaling is expected to continue down to the $k = 4$ node. This could lead to a capacitance as low as 500 aF. However, this is expected to be the end of the roadmap for scaling the photodiode size in terms of maintaining a reasonable quantum efficiency. From this perspective, the low capacitance phototransistor is expected to play a key role in overcoming the end of roadmap barrier in terms of reducing the link energy, by as much as an order of magnitude – or three scaling nodes – below the expected energy achievable with the receiverless approach.

VI. Prospects for silicon photonics integration

A major advantage of the low temperature growth of nanopillars is the possibility of back-end integration with CMOS silicon electronics. We have demonstrated growth on silicon transistors,

with optically pumped lasing of the nanopillars, along with un-degraded operation of the transistors after going through the MOCVD growth process (*Lu et al.,* Ref. 43 in the main text).

In addition, growth of InP nanopillars with InGaAs active region has been recently accomplished on a silicon-on-insulator platform. Waveguides have been patterned using e-beam lithography combined with a dry etch process based on an SF_6+O_2 plasma that selectively etches the silicon, and does not affect the InP-based nanopillars. Further, InP/InGaAs/InP multiple quantum well light emitting diodes have been demonstrated on chip at silicon transparent wavelengths of 1.3-1.55 μm (*Bhattacharya et al.,* Ref. 59 in main text). A first demonstration of waveguide coupling shows that the silicon-transparent emission of the nanopillars is coupled to the waveguide patterned at the base of the nanopillar (*Malheiros-Silveiras et al.,* Ref. 58 in main text). This will be the subject of future study, in order to improve sensitivity in highly scaled nanopillar structures with optimized coupling to waveguides. Relevant prior work on waveguide-nanophotodetector critical coupling to enhance photodetection in <100 aF capacitance devices includes that of *Going et al.,* Ref. 6 and Ref. 12 in the main text.

VII. High speed photodetector metrics

Here we briefly describe some important figures of merit for a photodetector device to be used as a sensitive and high speed receiver in an optical communication system. We have also compiled a table of reported devices based on InP phototransistors to clarify the contextual significance of our results, in reference to some of these metrics. This table corresponds to Fig. 1 in the manuscript.

Figures of merit:

Gain-bandwidth product: Equivalently the responsivity-bandwidth product, this is an important standalone figure of merit for high speed photodetectors. In general, there is a fundamental trade-off between the gain and the speed of a photodetector due to the device capacitance being amplified by the gain through the Miller effect (Ref. S6). It then becomes meaningful to compare the gain-bandwidth product across different kinds of photodetector devices – APDs, phototransistors, p-i-n diodes: with or without in-built gain, since the gain bandwidth product is

eventually limited by the capacitance. If the RC time is sufficiently low, carrier transit time sets the gain-bandwidth product limit. The transit time also sees a trade-off with absorption efficiency in a conventional top-illuminated geometry. A gain bandwidth product higher than 100 GHz is important for high speed and sensitive receivers. This requirement arises out of the need to provide a 10 dB sensitivity improvement while operating at a high speed of 10 GHz. The speed is usually set by system constrains and bandwidth demand.

Gain: In order to reach very low photon number operation in an optical link, it is essential to engineer some amplification or gain mechanism. For instance, this can be through either integrated transistor gain as described in this work, avalanche gain or photoconductive gain. Since the ultimate goal is to improve the sensitivity, it is required that the gain does not amplify the noise (excess noise figure). The gain element should ideally be placed physically close to the photodetection unit to avoid capacitance loading due to the connecting electrical wires. Gain usually also comes at the cost of high voltage bias (for the avalanche gain mechanism), or high current bias for transistor gain bandwidth product. Ideally, the gain would be available at CMOS line voltage, and would be engineerable through an electrical feedback mechanism so it is tolerant to temperature and device fluctuations. Circuit configurations for controllable gain and bias are well described for bipolar junction transistors (see for instance, Ref. S7 section 6.4 'Biasing in BJT circuits').

Dark current: The RF shot noise power is proportional to the absolute DC current of the device at the operating bias. Minimizing the dark current to a value well below the photocurrent expected under link operation is therefore essential. For 20 photons per bit at 10 Gb/s with a link operating at $1.55\ \mu m$, this corresponds to a photocurrent of approximately 32 nA. A dark current at the nA order or lower is therefore important in order to avoid dark current shot noise. This is particularly a concern for gap-less semiconductors such as graphene or nanoscale devices with relatively higher surface to volume ratio. In addition to bandgap, the dark current also depends on the material quality and surface passivation. We have earlier described (*Ko et al.*, Ref. 47 of main text) how the InP material system, combined with the core-shell growth mode leads to an

ultra-low dark current in a p-i-n junction. The measured dark current was ~1 fA for a device with 10 μm^2 area, well below the ~nA requirement.

External quantum efficiency/Absorption efficiency: This metric describes the number of photons that are captured by the device, without including the gain or amplification mechanism. For an energy efficient optical link, it is important to avoid wasting photons – which leads to requirements for a thick absorbing region with dimensions at least on the order of the diffraction limit for efficient light coupling. On the other hand, a thick absorbing region leads to a longer carrier transit time – leading to a trade-off between EQE and speed. Thus, it becomes important to use an absorber with a large absorption coefficient – such as direct bandgap compound semiconductors. Another approach to circumvent this trade-off is to use a waveguide integrated (S8, S10) or a nanoresonator geometry with a cavity or resonance effect (S1, S9, S10, S11). Waveguide integration decouples the transit direction from the absorption direction, but it also increases the perpendicular dimension of the device, leading to a larger capacitance.

Table 3: Comparison with relevant photodetectors for high speed and sensitive photodetection (focus on InP material system)

Year	Ref.	Electronic Gain-band width product f_T	Device technology details	Emitter-base junction size (or device size in general)	Collector current (DC bias), dark current where available	EQE (absorption efficiency)	Optical gain-bandwidth ($EQE \times f_T$)
This work		375 GHz	**Silicon substrate**, as-grown InP homojunction BJT with nano-resonator geometry	Core-shell junction, 0.5 μm radius, 1 μm height	Pulsed operation with ~1.8 fJ energy per pulse received at device	28%	**105 GHz**

2012	[18]	120 GHz	Ge lateral p-i-n, waveguide butt coupled	$\mathbf{500\ nm \times 170\ nm \times 10\ \mu m\ (length)}$	2 V bias for RF measurement, 4 μA dark current (at -1V)	66% (at 1550 nm)	**79 GHz**
2009	[17]	40 GHz	Ge lateral MSM, waveguide integrated	$\mathbf{1.5\ \mu m \times 260\ nm \times 30\ \mu m\ (length)}$	5 V bias, 4 μA dark current	90% (below 1540 nm)	**36 GHz**
1999	[5]	62 GHz	InP substrate, InGaAs absorber (HPT)	$4 \times 3.5\ \mu m^2$	10.7 mA	52.5%	**32.6 GHz**
2011	[19]	45 GHz	Ge vertical p-i-n, waveguide integrated	$\mathbf{1.3\ \mu m \times 600\ nm \times 4\ \mu m\ (length)}$	1 V bias, 3 nA dark current	65% (at 1530 nm)	**29 GHz**
1993	[2]	52 GHz	InP substrate, InGaAs absorber, 1.5 μm (HPT)	$3 \times 8\ \mu m^2$	5 mA	52%	**27.0 GHz**
2006	[13]	47 GHz	SiGe **BiCMOS** technology (250 nm node)	$2\ \mu m \times 2\ \mu m$	~0.1-0.2 mA collector current	393% including current gain	**21 GHz**
2015	[20]	35 GHz	Ge lateral p-i-n, waveguide integrated	$\mathbf{500\ nm \times 180\ nm \times 32\ \mu m\ (length)}$	5 V bias, 6 nA dark current	56% (at 1550 nm)	**20 GHz**
2006	[8]	447 GHz	InP substrate, InP/InGaAs double heterostructure, 1.55 μm, waveguide coupled (HPT)	$0.7 \times 4\ \mu m^2$	0.65 mA base current	4%	**17.9 GHz**

2000	[6]	82 GHz	InP substrate, InP/InGaAs double heterostucture, 1.55 μm (HPT)	24 μm^2	20 mA	20%	**16.4 GHz**
1993	[3]	30 GHz	InP substrate, InGaAs absorber, Edge-coupled (HPT)	$5 \times 10\ \mu m^2$	8 mA	50%	**15.0 GHz**
2014	[12]	99 GHz	**Silicon substrate**, as-grown InGaAs nanopillar homojunction APD	Core-shell junction, 0.5 μm radius, 1 μm height	-4.4 V bias	15%	**14.85 GHz**
2004	[7]	135 GHz	InP substrate, InP/InGaAs double heterostructure, 1.55 μm (HPT)	$2.6 \times 7.8\ \mu m$	20 mA	6.4%	**8.6 GHz**
2007	[10]	6 GHZ	Transferred ZnO nanowires after CVD growth, photoconductive gain	150-300 nm diameter, 10-15 μm length	5 V bias	Not reported	**6 GHz**
1994	[4]	14 GHz	InP substrate, InGaAs absorber, with back reflector (HPT)	$3 \times 3\ \mu m^2$	14 mA	21%	**2.8 GHz**
2008	[11]	11.4 GHz (14 ps FWHM)	Ensemble InP nanowires on amorphous SiO_2	~10 μm size ensemble	5 V bias	0.3 A/W	**2.1 GHz**

1981	[1]	1.7 GHz	InP substrate, InGaAs absorber , 1.154 μm (HPT)	10 μm radius	0.5 mA	70%	**1.19 GHz**
2013	[16]	18 GHz	2-3 layer graphene	24 μm waveguide integrated	-	4% (50 mA/W responsivity)	**0.72 GHz**
2006	[9]	6 GHz*	Fluid directed assembly of CdS/Si heterojunction nanowire APD	50-100 nm diameter, crossed junction	-8 V bias	0.1%	**0.06 GHz**
2015	[14]	3 GHz	11.5 nm multilayer black phosphorus (2D material)	6.5 μm length waveguide integrated device	Gate voltage of -8 V (doping), bias voltage of 0.4 V	1.5% (18.8 mA/W responsivity)	**0.045 GHz**
2009	[15]	40 GHz	2-3 layer graphene	3 μm^2 area (top-down illumination)	80 V Gate bias	0.04% (0.5 mA/W responsivity)	**0.016 GHz**

Color key:

Nanopillar/Nanowire Devices
Germanium or SiGe on a Silicon substrate
InP/InGaAs on an InP substrate
2D material (waveguide integrated)

From the above table, there are a few key takeaways. Firstly, devices with ultrahigh electronic transition frequency f_T have recently been engineered by scaling dimensions down and using a thin absorber (Refs. 5-8 below). But, this leads to a trade-off with the external quantum efficiency, thus degrading the $EQE \times f_T$ product (equivalent to a responsivity-bandwidth product –rightmost column). In the NPT, a nano-resonator geometry with a gold back-reflector (which doubles as an electrical contact) has been used to maintain absorption while keeping device size small. In addition, this is the first time that a high crystalline quality InP-based phototransistor has been monolithically integrated with Silicon at a low growth temperature, thus leading to the possibility of leveraging the Silicon photonics infrastructure and a close integration of further receiver electronics on a CMOS platform. Other nanowire work has focused on nanowires transferred to an insulating substrate after growth, instead of an as-grown device integrated on a scalable substrate. We have also included Germanium based photodetectors for WDM C-band operation, which is the most mature technology available (representative work: Ref. 17-20 below). The absorption around 1550 nm is rather low, which limits the extent to which the active region can be shrunk. SiGe Bi-CMOS (Ref. 13 below) is a relevant technology for heterojunction phototransistors, again with a caveat of low absorption, especially for low Germanium content. Lastly, 2D material photodetectors with waveguide integration have been demonstrated. The absorption is somewhat low due to the thin absorber, therefore necessitating a rather large device size (Ref. 14-16 below).

Photodetector table references:

[1] Campbell, J. C., Burrus, C. A., Dentai, A. G. & Ogawa, K. Small-area high-speed InP/InGaAs phototransistor, *Appl. Phys. Lett.* **39**, 820 (1981)

[2] Chandrashekar, S., Lunardi, L. M., Gnauck, A. H., Hamm, R. A. & Qua, G. J. High-speed monolithic p-i-n/HBT and HPT/HBTphotoreceivers implemented with simple phototransistor structure, *IEEE Photonic Tech. L.* **5**, 11 (1993)

[3] Wake, D; Newson, D. J.; Harlow, M. J.; Henning, I. D. Optically-biased, edge-coupled InP/InGaAs heterojunction phototransistors, *Electron Lett.*, **29**, 25, 2217-2219 (1993)

[4] Fukano, H.; Takanashi, Y.; Fujimoto, M. High-speed InP/InGaAs heterojunction phototransistors employing a nonalloyed electrode metal as a reflector, *IEEE J. Quant. Electron.*, **30**, 12 (1994)

[5] Thuret, J.; Gonzalez, C.; Benchimol, J. L.; Riet M.; Berdaguer, P. High-speed InP/InGaAs heterojunction phototransistor for millimetre-wave fibre radio communications, in *11th International Conference on Indium Phosphide and Related Materials*, Davos, Switzerland, 1999

[6] Kamitsuna, H., Matsuoka, Y., Yamahata, S . & Shigekawa, N. A 82-GHz-Optical-Gain-Cutoff-Frequency InP/InGaAs double-heterostructure phototransistor (DHPT) and its application to a 40-GHz-band OEMMIC photoreceiver, in *Microwave Conference, 2000. 30th European* (2000)

[7] Leven, A., Houtsma, V., Kopf, R., Baeyens, Y., Chen, Y.-K. InP-based double-heterostructure phototransistors with 135 GHz optical-gain cutoff frequency, *Electron. Lett.*, **40**, 13, 2004

[8] Houtsma, V. E., Leven, A., Chen, J., Frackoviack, J., Tate, A., Weimann, N. G. & Chen, Y.-K. High gain-bandwidth InP waveguide phototransistor, *Indium Phosphide and Related Materials Conference Proceedings, International Conference on*, 247-251, 7-11 May 2006

[9] Hayden, O., Agarwal, R. & Lieber, C. M. Nanoscale avalanche photodiodes for highly sensitive and spatially resolved photon detection, *Nat. Mater.*, **5**, 352-356, 2006

[10] Soci, C., Zhang, A., Xiang, B., Dayeh, S. A., Aplin, D. P. R., Park, J., Bao, X. Y., Lo, Y. H. & Wang, D. ZnO nanowire UV photodetectors with high internal gain, *Nano Lett.*, **7** (4), 1003-1009, 2004

[11] Logeeswaran, V. J., Sarkar, A., Islam, M. S., Kobayashi, N. P., Straznicky, J., Li, X., Wu, W., Mathai, S., Tan, M. R. T., Wang, S-H. & Williams, R. S., A 14-ps full width at half maximum high-speed photoconductor fabricated with intersecting InP nanowires on an amorphous surface, *Appl. Phys. A*, **91**, 1-5, 2008

[12] Chen, R., Ng, K.-W., Ko, W. S., Parekh, D., Lu, F., Tran, T.-T. D., Li, K. & Chang-Hasnain, C. J., Nanophotonic integrated circuits from nanoresonators grown on silicon, *Nat. Comm.*, **5**, 4325, 2014

[13] Yin, T., Pappu, A. M. & Apsel, A. B., Low-cost, high-efficiency, and high-speed SiGe phototransistors in commercial BiCMOS, *IEEE Photon. Technol. Lett.*, **18**, 1, 2006

[14] Youngblood, N., Chen, C., Koester, S. J., & Li, M., Waveguide-integrated black phosphorus photodetector with high responsivity and low dark current, *Nat. Photonics*, **9**, 247, 2012

[15] Xia, F., Mueller, T., Lin, Y.-M., Valdes-Garcia, A., & Avouris, P., Ultrafast graphene photodetector, *Nat. Nanotechnology,* **4**, 839, 2009

[16] Popischil, A., Humer, M., Furchi, M. M., Bachmann, D., Guider, R., Fromherz, T., & Mueller, T., CMOS-compatible graphene photodetector covering all optical communication bands, *Nat. Photonics,* **7**, 892, 2013

[17] Chen, L., & Lipson, M., Ultra-low capacitance and high speed germanium photodetectors on silicon, *Opt. Express,* **17**, 10, 2009

[18] Vivien, L., Polzer, A., Marris-Morini, D., Osmond, J., Hartmann, J. M., Crozat, P., Cassan, E., Kopp, C., Zimmerman, H., & Fédéli, J. M., Zero-bias 40 Gb/s germanium waveguide photodetector on silicon, *Opt. Express,* **20**, 2, 2012

[19] DeRose, C. T., Trotter, D. C., Zortman, W. A., Starbck, A. L., Fisher, M., Watts, M. R., & Davids, P. S., Ultra compact 45 GHz CMOS compatible Germanium waveguide photodiode with low dark current, *Opt. Express*, **19**, 25, 2011

[20] Going, R., Seok, T. J., Loo, J., Hsu, K., & Wu, M. C., Germanium wrap-around photodetectors on Silicon photonics, *Opt. Express*, **23**, 9, 2015

Supplementary information text references:

[S1] Ko, W. S., Tran, T.-T. D., Bhattacharya, I., Ng, K. W., Sun, H. & Chang-Hasnain C. J., Illumination angle insensitive single indium phosphide tapered nanopillar solar cell, *Nano Lett.*, **15** (8), 4961-4967, 2015

[S2] Ren, F., Ng, K. W., Li, K., Sun, H. & Chang-Hasnain, C. J. High-quality InP nanoneedles grown on silicon, *Appl. Phys. Lett.*, **102**, 012115, 2013

[S3] Hu, C., *Modern semiconductor devices for integrated circuits* (Prentice Hall 2010)

[S4] Sze, S. M., *Physics of semiconductor devices* (Wiley, 2nd edition, 1981)

[S5] Li, K., Ng, K. W., Tran, T.-T. D., Sun, H., Lu, F. & Chang-Hasnain C. J. Wutzite-phased InP micropillars grown on silicon with low surface recombination velocity, *Nano Lett.*, **15**(11), 7189-7198, 2015

[S6] Miller, J. M., Dependence of the input impedance of a three-electrode vacuum tube upon the load in the plate circuit, *Scientific Papers of the Bureau of Standards*, **15** (351): 367-385, 1920

[S7] Sedra, A. S., Smith, K. C., *Microelectronic circuits* (3rd edition, The Holt, Rinehart and Winston series in electrical engineering, Saunders college publishing, 1990)

[S8] Michel, J., Liu, J. & Kimmerling, L. C. High-performance Ge-on-Si photodetectors, *Nat. Photonics*, **4**, 527-534, 2010

[S9] Tran, T.-T. D., Chen, R., Ng, K. W., Ko, W. S., Lu, F. & Chang-Hasnain, C. J. Three-dimensional whispering gallery modes in InGaAs nanoneedle lasers on silicon, *Appl. Phys. Lett.*, **105**, 111105, 2014

[S10] Going, R., Kim, M.-K. & Wu, M. C. Metal optic cavity for a high efficiency sub-fF Germanium photodiode on a silicon waveguide, *Opt. Express*, **21** (19), 22429-22440, 2013

[S11] Cao, L., Fan, P., Vasudev, A. P., White, J. S., Yu, Z., Cai, W., Schuller, J. A., Fan, S. & Brongersma, M. L. Semiconductor nanowire optical antenna solar absorbers, *Nano Lett.*, **10** (2), 439-445, 2010
